# The underlying mechanism of physical exercise on neurodegenerative diseases: the mediating role of psychological stress and resilience: a systematic review

**DOI:** 10.3389/fpsyg.2025.1474579

**Published:** 2025-07-14

**Authors:** Qingxia Jiang, Yan Liu, Yong Wei, Garry Kuan, Lei Ma, Huarong Wang, Yaxian Wang, Hu Lou

**Affiliations:** ^1^School of Sports Science, Nantong University, Nantong, China; ^2^Institute of Rehabilitation Research, Nantong Rehabilitation Hospital, Nantong, China; ^3^School of Health Sciences, Universiti Sains Malaysia, Kubang Kerian, Kelantan, Malaysia; ^4^School of Information Science and Technology, Nantong University, Nantong, China; ^5^Institute of Special Environmental Medicine, Nantong University, Nantong, China; ^6^Nursing College of Nantong University, Nantong University, Nantong, China

**Keywords:** physical exercise, stress, resilience, neurodegenerative disease, Alzheimer's disease, Parkinson's disease

## Abstract

Theories and experiments have shown that physical exercise can improve mental resilience and resist the negative effects of psychological stress. Neurodegenerative diseases are a collection of diseases in which progressive changes in the structure and function of neurons result in progressive disorders of cognitive and motor function, greatly reducing the quality of life of the patient. The latest research suggests that psychological factors such as psychological stress and resilience also have an impact on the onset, symptoms, and course of Neurodegenerative diseases. However, the specific mechanisms in the above pathways are unclear, so this study introduced psychological factors such as psychological stress and resilience and explore the mechanism of physical exercise in improving NDDs by influencing psychological factors such as psychological stress and resilience. This review examined four electronic databases (PubMed, Web of Science, Cochrane Library, and CNKI) up to May 2024, selecting a total of 95 articles. A logical analysis approach was employed to evaluate the literature. The findings revealed that: (1) Exercise can enhance resilience by reducing negative emotions or fulfilling individual needs, thereby diminishing the harmful effects of stress, a key risk factor for NDDs. (2) Exercise alleviates NDDs through neurobiological pathways such as improving immune function, regulating endocrine and neurotransmitter levels, and modifying neuronal structure. (3) Long-term, regular high-intensity exercise effectively enhances resilience and helps prevent and treat NDDs. Exercise has a positive impact on the prevention and treatment of NDDs. Clarifying the mechanisms by which exercise improves NDDs is crucial for providing new theoretical insights into the diagnosis, prevention, and treatment of psychologically induced NDDs, as well as offering practical guidance and feasible strategies for using exercise to prevent and mitigate NDDs.

## 1 Introduction

Neurodegenerative diseases (NDDs) are a group of disorders characterized by neuronal damage and death, including Alzheimer's Disease (AD), Parkinson's Disease (PD) and Huntington's Disease (HD), Amyotrophic Lateral Sclerosis (ALS), Motor Neuron Disease (MND), and other diseases. NDDs are often accompanied by symptoms such as cognitive decline, movement disorders, and mental behavioral abnormalities, which have a serious impact on the quality of life of patients and greatly reduce their quality of life (Kok et al., 2022). The accelerating global population aging has precipitated a rising prevalence of NDDs among older adults, underscoring an urgent need to develop effective countermeasures (Zatková et al., 2024).

Much of the established theoretical research and practice has focused on the various neurobiological factors that lead to NDDs, however, recent studies have found that psychological factors such as psychosocial stress are also associated with the pathogenic disease process of NDDs (Vyas et al., 2016). Currently, the treatment of NDDs disease mainly relies on Western single molecular targeted therapy and emerging Chinese medicine. However, clinical trials of single molecule-targeted drugs have mostly failed due to adverse reactions during trials, poor drug specificity, and lack of efficacy (Zhang et al., 2023). Known for its ability to treat diseases before they occur, traditional Chinese medicine with its advantages of early treatment before illness, holistic theory, integration concept, and two-way regulation theory, holds great promise, but there are no particularly effective results yet (Han et al., 2021). Both types of treatments have yet to make a breakthrough, and the research is temporarily stuck in a bottleneck. Physical activity has been proposed to prevent and alleviate NDDs mediated by various psychological, with the advantages of low cost and few side effects. However, the mechanisms of how physical activity affects cognitive impairment, susceptibility, and disease course in patients with psychogenic NDDs are unclear. Therefore, this study innovatively introduced psychological stress and resilience, and based on the pathogenesis of NDDS, explored how psychological factors such as psychological stress and resilience affect the onset, symptoms, and disease course of patients with NDDs as well as the mechanism of physical exercise improving the cardiogenic neurodegenerative diseases, and gave the corresponding reference for physical exercise, to provide new theoretical reference. It also provides practical references and feasible solutions for the prevention and alleviation of neurodegenerative diseases through physical exercise.

## 2 Materials and methods

**Figure 1:**
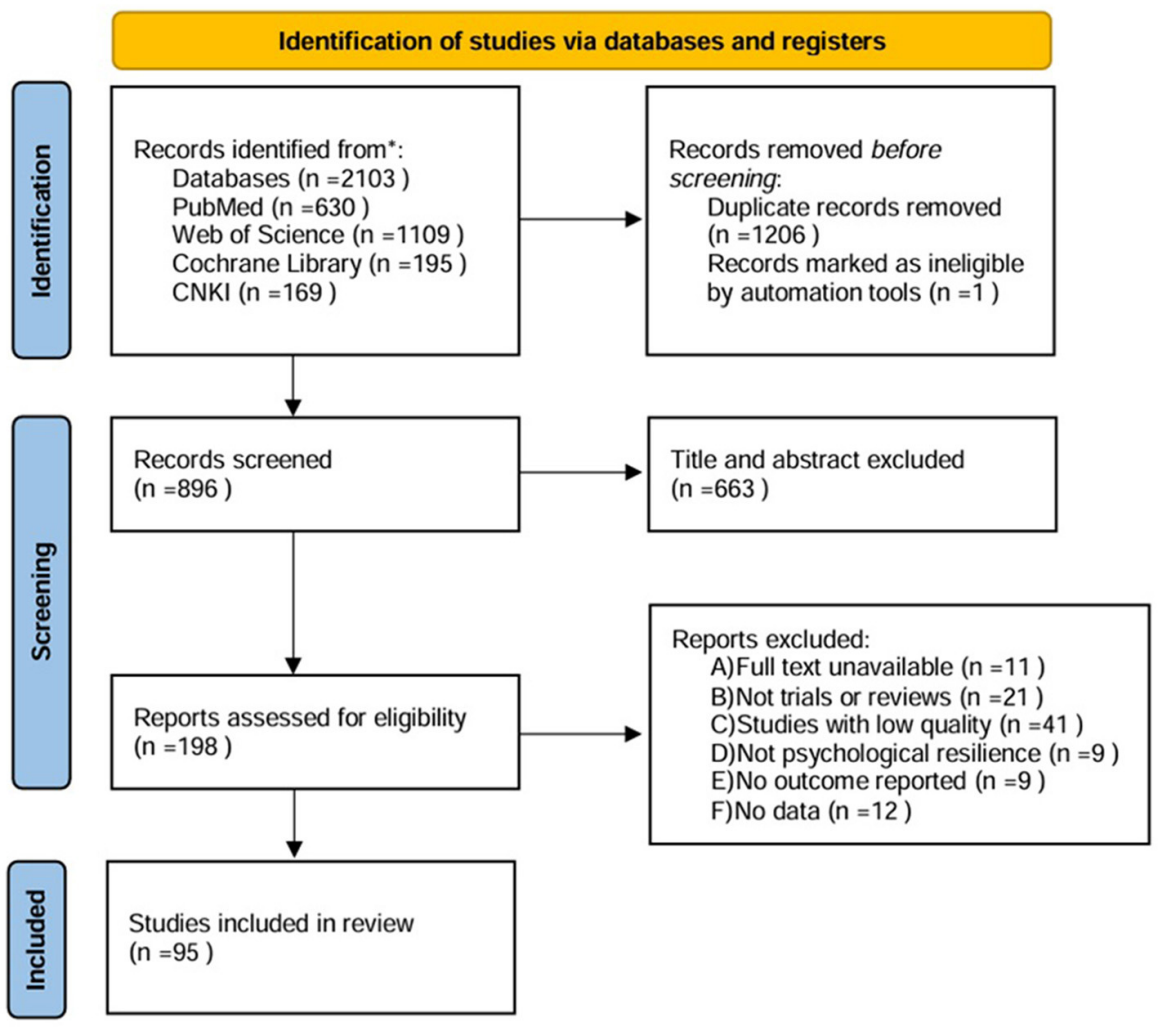
PRISMA 2020 flow diagram.

PubMed, Web of Science, Cochrane Library, and the China National Knowledge Infrastructure (CNKI) were searched from the inception of each database to 1 May 2024. Searches were conducted using MeSH descriptors: Neurodegenerative diseases, Alzheimer's disease, Parkinson's disease, stress, resilience, and exercise..., with Boolean operators AND and OR. No language limit was applied. Two independent reviewers screened the title and abstract of the retrieved articles, and the full text was reviewed as necessary. The studies that were potentially relevant according to the eligibility criteria were selected. Disagreements regarding study inclusion were resolved by discussion, and in cases of persistent disagreement, a third reviewer was consulted. The study selection process was visualized using the Preferred Reporting Items for Systematic Reviews and Meta-Analyses (PRISMA) diagram. Inclusion Criteria: Studies investigating the impact and mechanisms of psychological stress and/or psychological resilience on NDDs (e.g., Parkinson's disease, Alzheimer's disease), as well as studies examining the effects and mechanisms of various types of physical exercise on NDDs. Exclusion Criteria: Duplicate publications, retracted articles, conference abstracts, letters, or articles lacking original data; inaccessible full texts; studies without results or data; articles irrelevant to the research objectives; or studies failing to meet the inclusion criteria. The PRISMA diagram is shown in Figure 1.

## 3 The neurobiological pathogenesis of NDDs

With the development of research, the understanding of the pathogenesis of NDDs is getting deeper and deeper, and the neurobiological pathological causes are Ca2+ imbalance, apoptosis, aging, abnormal gene regulation and brain metabolic disorders, etc., and the main pathogenetic mechanisms are oxidative stress, immune- inflammatory, excitotoxicity and mitochondrial function mechanisms (Xue et al., 2015).

### 3.1 Mechanisms of oxidative stress

In the Central Nervous System (CNS) of the human brain, if oxygen free radicals are overproduced and/or not scavenged in a timely manner, oxidative stress can damage cells and tissues in the brain, resulting in cerebrovascular dysfunction, peroxidative cellular damage, and even apoptosis, which in turn leads to NDDs.

Oxygen free radicals are mainly caused by excitatory amino acids, metabolism of neurotransmitters, and mitochondrial dysfunction or perturbation of the Electron Transfer Chain (ETC) due to mitochondrial damage, which results in the production of large amounts of Reactive Oxygen Species (ROS) by mitochondria (Wilkaniec et al., 2021). The Mitochondrial ROS (mtROS) are produced by mitochondrial complex III and complex I, and their levels increase in response to cellular stress as well as perturbations in the electron transport chain. mtROS is generated when some of the electrons are transferred directly into O2 in the IF and IQ sites of complex I, the IIF site of complex II, and the IIIQo site of complex III (Pedrinolla et al., 2017). CMS Collaboration et al. (2015) found diminished mitochondrial respiratory chain complex I function with increased ROS and enhanced antioxidant enzyme activity in patients with maternally inherited familial PD. In addition, enhanced lipid peroxidation, reduced GSH consumption, and increased oxidative stress were found in the SNpc.

Following ROS production by mitochondria, oxygen free radicals can activate the structural domain of pyrin, a multiprotein complex formed by caspase-1 zymogen and ASC in the inflammatory vesicle NLRP3, cleaving and releasing mature forms of the Proinflammatory cytokines IL-1 βand IL-18 (McClelland et al., 1991), causing neurological inflammation and damage to brain cellular tissue, which in turn induces or accelerates the course of NDDs (Chen and Zhong, 2014).

### 3.2 Mechanisms of mitochondrial function

Mitochondria are the only organelles in the human body that contain extra-nuclear genetic material (mtDNA) that converts carbohydrates, proteins, and lipids into CO_2_ and water, generating large quantities of adenosine triphosphate (ATP), which provides energy for the cell. ROS is a key signal in the regulation of the activation of the NLRP3 inflammatory vesicles, and it has been shown that mtDNA also induces the production of ROS (McClelland et al., 1991), thereby exacerbating NDDs.

AD patients have mtDNA defects and oxidative phosphorylation abnormalities in the brain. The dysfunction of neuronal mitochondria in AD patients will lead to insufficient neuronal energy supply and release of a large amount of ROS, which induces oxidative stress injury, imbalance of calcium regulation, activation of inflammatory vesicles NLRP3, accumulation of neurotoxicity, and ultimately triggers neuronal apoptosis, accelerating the course of NDDs (Van Der Walt et al., 2003).

However, some scholars believe that the activation of NLRP3 may not be dependent on ROS, and that ROS, which increases with NLRP3 activation, may only be triggered by mtDNA, an activator of NLRP3. There is still some controversy about whether ROS can activate NLRP3 and thus lead to NDDs.

### 3.3 Mechanisms of Excitotoxic

As the most important excitatory neurotransmitter in the mammalian CNS, normal concentrations of glutamate regulate normal physiological functions of the CNS such as neurotransmitter release, synaptic plasticity, and synaptic long-range potentiation and inhibition. However high levels of glutamate have excitotoxic effects and are associated with a variety of neurodegenerative diseases such as multiple sclerosis, Alzheimer's disease, and amyotrophic lateral sclerosis (Rothman, 1985).

When β-amyloid precursor proteins and/or Tau proteins inhibit extracellular glutamate uptake and glutamate concentration in the cellular interstitial space is too high, it leads to neurotoxicity in neurons, e.g., Ca2+ endocytosis mediated by overexcitation of the NMDA receptors causes delayed injury of nerve cells; Na+, Cl-, and water endocytosis mediated by overexcitation of the AMPA receptors and KA receptors cause acute osmotic swelling, which ultimately leads to degeneration, senescence, and death of brain neurons that cannot regenerate and catalyzes the onset and development of NDDs (Xue et al., 2015).

### 3.4 Mechanisms of immuno-inflammatory

Mechanistic studies of NDDs have focused on appealing several pathways, and in recent years, attention has been focused on the relationship between inflammation and NDDs, and the inflammatory hypothesis is becoming a research hotspot for the pathogenesis of NDDs (Huang and Zhou, 2021).

NDDs is an umbrella term for a group of diseases, but they share many common and diverse pathological and clinical features, including selective susceptibility of brain regions and aggregation of different proteins, and persistent chronic inflammation (Zhang et al., 2023). The long-term persistence of chronic neuroinflammation is closely associated with NDDs, and neuroinflammation in patients with NDDs is caused by a translational imbalance of different microglia morphologies in the central nervous system. Immunity is bi-directional, and as part of the immune system, microglia have two functionally different activation states, M1 and M2, where M2 microglia sense and internalize the removal of misfolded proteins, release anti- inflammatory factors such as IL-4 and IL-13 to remove inflammation and pathogens, maintain neural tissue homeostasis, and repair damage. After activation, M1 microglia release inflammatory factors such as IL-1β and IL-18 produced by NLRP3 inflammasome into the extracellular environment to form an inflammatory environment (Cell Signaling Technology, 2023). Under normal conditions, M1 and M2 microglia are in a dynamic equilibrium and work together to regulate programmed neuronal cell death, strip excess synapses from developing neurons, promote synapse formation, and protect the CNS from excessive neuroinflammatory and immune response damage (Han et al., 2021). However, M2 microglia in the CNS of patients with NDDs are suppressed, and M1 microglia are over-activated for a long period and proliferate in large numbers, continuously releasing inflammatory factors, leading to a long-term chronic inflammatory environment for the nerves in the brain, resulting in β-amyloid (Aβ), Neurofibrillary tangle (NFT), and NFTs in the CNS (Li et al., 2022) massive accumulation and neurotoxicity (Duggan and Parikh, 2021) accumulation, ultimately leading to neuronal tissue degeneration, impaired glial stress and cognitive decline (Li et al., 2022; Zhang et al., 2023). This microglia- mediated neuroinflammation leads to neuronal damage, activating M1 microglia, creating a vicious cycle and accelerating the disease process in NDDs (Zou et al., 2023).

Through animal as well as human experiments, researchers have validated the immunoinflammatory theory of pathogenesis. Cicchetti et al. (2002) observed a parallel relationship between the loss of dopaminergic neurons and the activation of microglia at this site in an animal model of PD. Mcgeer and Rogers (1992) found that long-term use of non-steroidal anti-inflammatory drugs attenuated the pathogenesis of AD patients.

## 4 Mechanisms by which psychological factors such as stress and mental toughness influence NDDs

**Figure 2:**
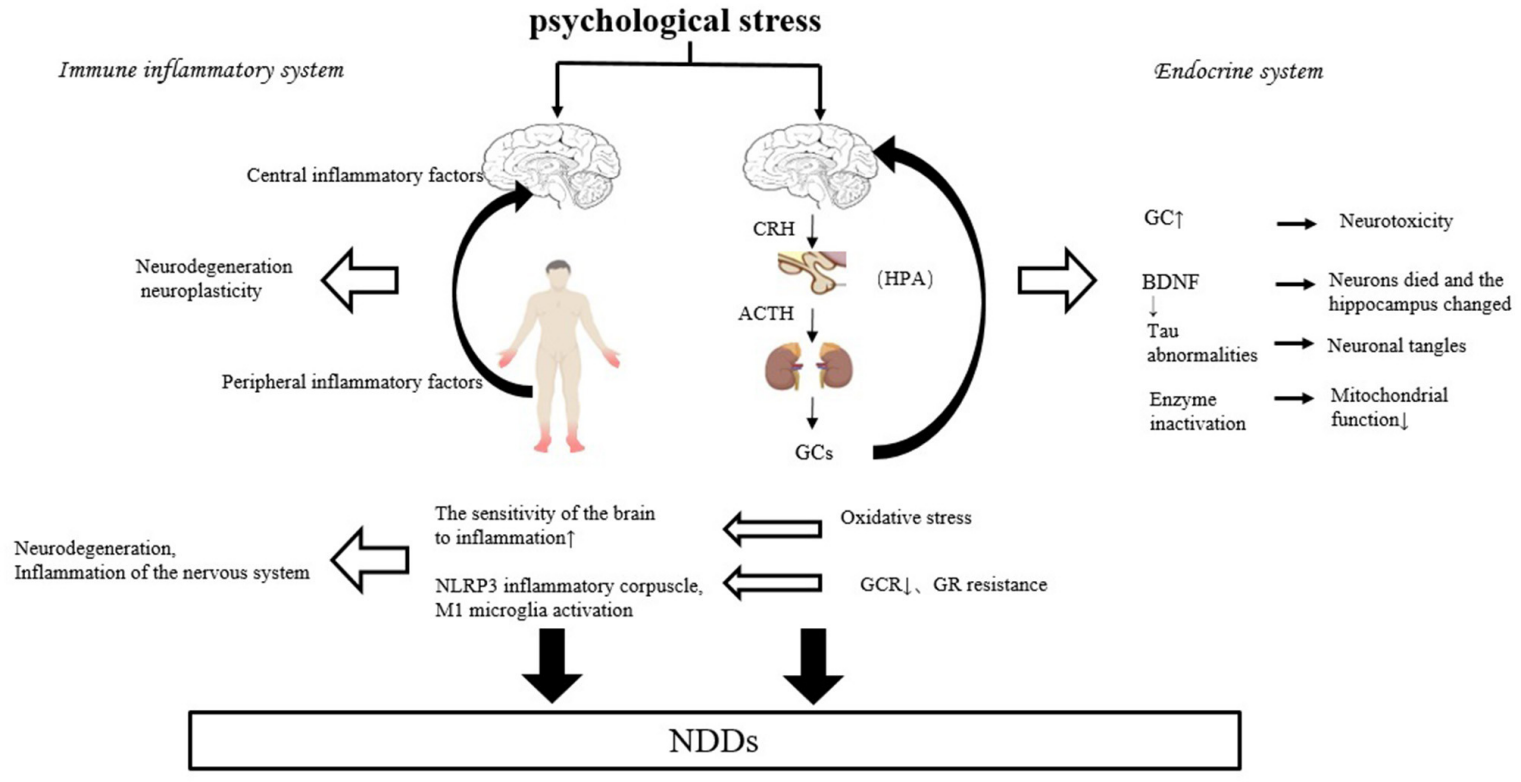
Possible mechanisms of psychological stress on the negative effects of NDDs.

There is growing evidence of a link between chronic psychological stress and neurodegenerative diseases such as AD and PD (Vyas et al., 2016). Vyas et al. (2016) and other Scholars believe that individuals suffering from high psychological stress early in life increase their susceptibility to NDDs under the mediating effect of high glucocorticoid (GC). Machado et al. (2014) analyzed that chronic psychological stress may increase the incidence of AD and/or accelerate AD onset, and disease progression (Vyas et al., 2016), thus chronic psychological stress is a risk factor for NDDs such as AD. Psychological stress can negatively affect the occurrence and progression of NDDs, mainly through the endocrine humoral system and immune-inflammatory pathways (Figure 2).

Exposure to stressful events is common in modern society (Lou and Yan, 2020). The psychological stress experienced by patients, especially after developing NDDs, is immense, however, Majnarić et al. (2021) found that when an individual has difficulty overcoming excessive psychological stress, the homogeneous load triggers a series of pathological and physiological responses in the body systems and organs, increasing the susceptibility and severity of NDDs in that individual. Therefore, individuals need to maintain positive adaptability in the face of stress. Babić et al. (2020) and other scholars found that the higher the mental toughness of an individual, the lower the susceptibility to NDDs and the risk of developing the disease. Mental toughness can modulate an individual's resistance and/or adaptability to psychological stress through various psychological and neurobiological mechanisms (Qiu et al., 2023).

### 4.1 Possible mechanisms for the negative effects of psychological stress on NDDs

#### 4.1.1 Endocrine humoral system

Psychological stress can be perceived by The Hypothalamic-pituitary-adrenal Axis (HPA). When an individual suffers from psychological stress, the hypothalamus first secretes Corticotropin-Releasing Hormone (CRH), which triggers the pituitary gland to release adrenocorticotropic hormone (ACTH) into the bloodstream, inducing the secretion of cortisol from the adrenal cortex. Machado demonstrated in 1991 that plasma cortisol increased following the experience of psychological stress. One of the most easily measured and critical physiological responses to psychological stress is GC (Machado et al., 2014).

Machado et al. (2014) believe that psychological stress can increase extracellular glutamate accumulation via GC, and the accumulation of large amounts of glutamate can exert neurotoxic effects, leading to or exacerbating NDDs. GC can also play a negative role in the pathogenesis of NDDs by triggering apoptosis and cell death. Psychological stress may also induce neuronal atrophy and synaptic dysfunction or loss through GC-stimulated hyperphosphorylation of the cytoskeletal protein Tau, which disrupts cytoskeletal integrity and mismatches Tau at synapses, ultimately leading to the degradation of synaptic proteins and receptors, thereby blocking synaptic transmission in neurons (Vyas et al., 2016). GC also destabilizes microtubules by reducing the binding of Tau to them, causing proteins to aggregate and form neurogenic fiber tangles, exacerbating symptoms such as memory and cognitive impairment in patients with NDDs (Horowitz et al., 2013).

Glucocorticoid Receptor (GR) has been shown to play an important role in regulating the expression of Brain-derived Neurotrophic Factor (BDNF). However, the lack of BDNF may increase neuronal cell death, alter hippocampal neurogenesis, and thereby affect the onset and progression of NDDs (Horowitz et al., 2013).

Chronic psychological stress and low psychological resilience may also influence the course of NDDs through aging. Studies have shown that the inability to adapt to chronic psychological stress or prolonged exposure to chronic high GC levels accelerates senescence and reduces mitochondrial function through the inactivation of several enzymes or reduction of enzyme expression, reduces energy production, increases oxidative stress, reduces intracellular calcium buffering, enhances cellular vulnerability, and several other pathways that have an impact on the pathogenesis of NDDs, especially in brain regions that are subject to high GR concentrations, and which are affected by chronic psychological stress are more affected (Machado et al., 2014; Majnarić et al., 2021).

Psychological stress reduces the level of free radical scavenging and the activity of metabolic enzymes such as catalase, which may lead to increased levels of ROS after psychological stress (Machado et al., 2014), producing oxidative stress effects such as NLRP3 inflammatory vesicle activation and causing microglia to release the Proinflammatory cytokines IL-1β and IL-18 (McClelland et al., 1991), causing neurological inflammation and damage to brain cellular tissue (Xue et al., 2015), thereby inducing or accelerating the course of NDDs. Oxidative stress also induces Aβ formation by increasing the enzymes involved in Aβ production. As the rate of Aβ accumulation increases, Aβ, as one of the main activators of microglia, also increases the production of ROS, causing lipid peroxidation and protein oxidation, thus creating a vicious cycle (Wang et al., 2023). The accumulation of Aβ and neurogenic fiber tangles in the brains of patients in the cycle may lead to or exacerbate symptoms such as cognitive impairment in patients with NDDs.

#### 4.1.2 Immune-inflammatory system

There is a large body of research supporting the immuno-inflammatory hypothesis mechanism, and clinical studies have shown that microglia that release inflammatory markers during chronic psychological stress are activated in the prefrontal cortex (Walker et al., 2017). Animal experiments have shown that chronic psychological stress activates microglia and alters their density and morphology in rodents, especially in stress-sensitive brain regions such as the hippocampus, prefrontal cortex, and amygdala. It also shows that the negative effects of psychological stress have a long-tailed impact, with microglia activation persisting after the cessation of the psychological stress stimulus (Dudek et al., 2021). In addition, psychological stress increases central proinflammatory cytokines (Machado et al., 2014). For example, IL-1β is increased in the hippocampus after chronic mild psychological stress, and proinflammatory cytokines accelerate neurodegeneration, affect regional brain activity such as cortico-striatal-limbic circuits, and alter neuroplasticity (Horowitz et al., 2013), and thus influence the pathogenesis of NDDs. In addition, chronic psychological stress accelerates immune senescence, and with cellular senescence and immune system aging, there is increased production of Proinflammatory cytokines and increased levels of systemic inflammation, and these peripherally produced Proinflammatory cytokines, ultimately, flow back into the CNS, and contribute to long-term inflammation in the CNS (Majnarić et al., 2021).

Psychological stress may also enhance the sensitivity of brain regions to inflammation in patients with NDDs such as AD. The immune system's responsiveness to inflammatory compounds such as lipopolysaccharide (LPS) may increase under psychological stress. Chronic psychological stress was also found to exacerbate inflammation caused by proinflammatory compounds in the prefrontal cortex and hippocampus, and these two brain regions exhibited significantly increased inflammatory responses and neuronal degeneration (Machado et al., 2014).

GC is often considered anti-inflammatory and does have a variety of inhibitory effects on inflammation. While GC secretion increases under psychological stress, and GC may increase inflammatory responses under chronic psychological stress (Macpherson et al., 2005), as long-term chronic stress leads to reduced GR resistance and glucocorticoid signaling, thereby exacerbating inflammation in the relative absence of its normal inhibitory effects. A significant correlation was found between increased inflammatory signaling and reduced glucocorticoid signaling in subjects suffering from chronic psychological stress (Horowitz et al., 2013). Chronic high levels of GC impair immune function in part by down-regulating GR, changing the protective effects of GC to inflammatory effects and inducing apoptosis, neuronal damage, and brain lesions, thus affecting the course of NDDs. Prolonged exposure to high GC levels may also reduce GR sensitivity in the brain, leading to reduced negative feedback (Machado et al., 2014). Impaired negative feedback may lead to further long- term elevations in cortisol, thereby prolonging the inflammatory cycle (Gaffey et al., 2016) that leads to the development and progression of NDDs (Vyas et al., 2016). In addition, normally GR and NF-κβ signaling pathways inhibit each other at the protein and molecular level to form a long-term homeostasis equilibrium, but this equilibrium may be affected by chronic psychological stress. Decreased GR function after long-term psychological stress leads to a lack of transcriptional repression of NF-κβ. Whereas the transcription factor NF-κβ is critical for alterations in the relative expression of transcripts containing response elements in the immune system, and the expression of chemokines and cytokines following activation of cellular inflammation (Horowitz et al., 2013).

It is thought that there is an interaction between the endocrine system and the immune-inflammatory system. Cytokines in the immune-inflammatory system also affect the availability of cortisol in the endocrine system. Proinflammatory cytokines can activate the HPA axis, which can lead to HPA hyperactivity or GR resistance. Hyperactivity of the HPA axis can lead to abnormalities in the neuroendocrine system (Capuron et al., 2003). GCs in the endocrine system can affect neuronal structure and function by regulating neuroinflammatory processes such as microglia activation in the immune system and epigenetic mechanisms (De Pablos et al., 2006), generating a vicious cycle. Proinflammatory cytokines also directly impair various aspects of GR function, such as GR translocation from the nucleus, GR protein-protein interactions, and GR binding to response elements on DNA, causing impaired and resistant GR function and leading to dysfunctional glucocorticoid signaling (Pace and Miller, 2009), which in turn has an impact on NDDs.

However, it has also been suggested that the two do not interact. Glucocorticoids and inflammatory signaling do not inhibit each other under certain concentrations and temporal conditions, and this occurs in key parts of the brain, such as the hippocampus (Horowitz et al., 2013).

### 4.2 Possible mechanisms by which mental toughness can have a positive effect on NDDs

**Figure 3:**
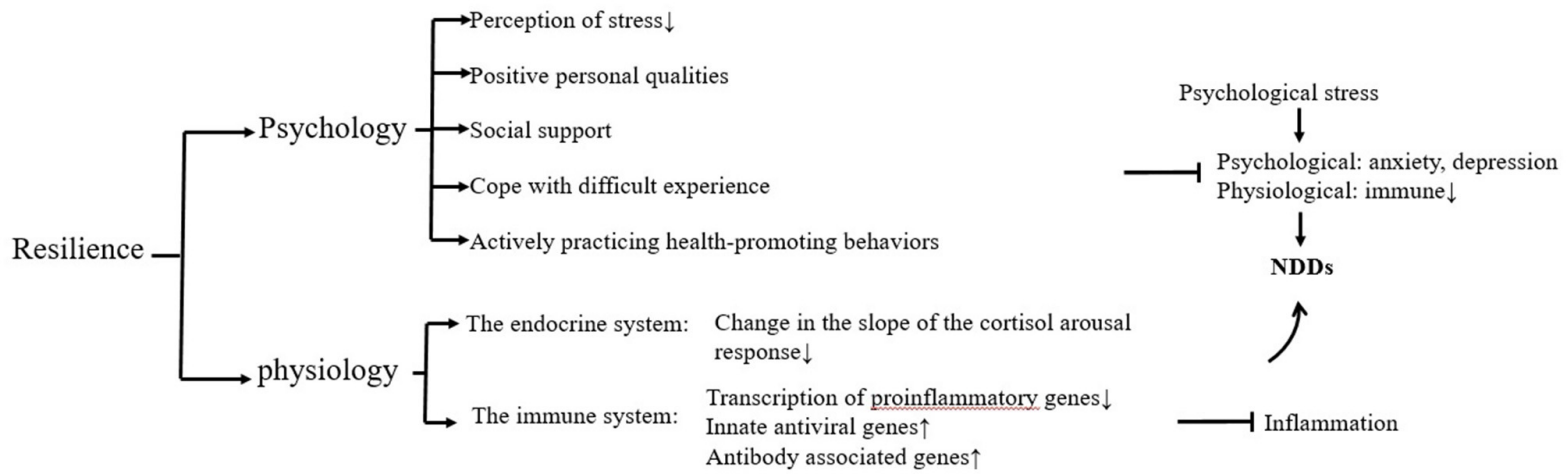
Possible mechanisms of resilience on the positive effects of NDDs.

Both chronic mental stress and mental toughness can affect an individual's health status (Gaffey et al., 2016) and thus have an impact on the course of disease in individuals with NDDs, with higher psychological resilience resulting in lower individual vulnerability and lower susceptibility to NDDs and risk of developing the disease (Babić et al., 2020). Resilience is an individual's ability to maintain or quickly return to normalcy in the face of adversity, trauma, tragedy, threat, or other significant stress. Lower mental toughness is associated with poorer cognition and a greater risk of dementia (Franks et al., 2023), and individuals with higher levels of mental toughness are more likely to cope with NDDs (Thomassen et al., 2018). As a result, some patients have a high quality of life and wellbeing despite having an NDDs or NDDs susceptibility, and studies have shown that patients with higher mental toughness have a prolonged period in which symptoms of cognitive impairment are not expressed, which greatly alleviates the condition and life distress of patients with NDDs (Kok et al., 2022; Neuner et al., 2022), and contributes to successful adaptation to NDDs disease. Possible mechanisms for the positive effects of mental toughness on NDDs can be explained psychologically and neurobiologically (Figure 3).

#### 4.2.1 Psychology

Higher mental toughness can reduce physiological responses to psychological stress by moderating an individual's perception of stressful situations (Majnarić et al., 2021). Through positive personal qualities, such as positive emotions and optimistic qualities, mental toughness can also enable individuals to adapt well to and recover from psychological stress, thereby preventing the harmful effects of psychological stress on physiological functioning, especially immunity. The reverse is also true, as there is evidence that immune processes also influence mental toughness (Dantzer et al., 2018). Patients with high mental toughness tend to have better emotional regulation in the face of stress and can maintain a better mood and calmer emotional responses, thus positively adapting to and transforming stressful dilemmas. They can also cope with anxiety and depression due to NDDs through optimistic qualities, seeing everything as a useful experience, focusing on personal strengths and qualities, and adopting constructive criticism, thus ultimately improving their quality of life (Jakovljevic and Jakovljevic, 2019).

Patients with high mental toughness tend to have better social support (Dantzer et al., 2018), and they tend to build close relationships with others, develop social skills, and have better emotional awareness. Thus, despite having NDDs or being at high risk of NDDs, they have relatively better cognitive functioning, higher quality of life, and wellbeing (Babić et al., 2020).

In addition, they have a wealth of experience in coping with difficulties and actively practice various health-promoting or health-maintaining behaviors such as access to screening tests and treatments, healthy eating, physical activity, and others (Majnarić et al., 2021). The main objective of this study is to ensure that people with NDDs successfully adapt to change or resist the negative effects of psychological stress and avoid significant dysfunction (Kok et al., 2022; Neuner et al., 2022).

#### 4.2.2 Neurobiology

Recent research has shown that differences in levels of mental toughness between individuals can be expressed through differences in the temporal dynamics of neurophysiological toughness (Watanabe and Takeda, 2022), such as stress hormones (HPA axis) and the immune system.

Mental toughness can act through the endocrine system, moderated by and/or interacting with social support and emotion regulation (Gaffey et al., 2016). High mental toughness populations show less variation in the slope of the cortisol arousal response (García-León et al., 2019). In addition, mental toughness can positively influence NDDs through immune system responses. Mental toughness scores were significantly and positively correlated with perceived health and perceived immune function. In human experiments, Van Schrojenstein Lantman et al. (2017) scholars found that mentally resilient individuals had better physical and mental health and better immune system functioning. In animal experiments, Watanabe and Takeda (2022) found that mice with lower mental toughness had compromised immune system cell numbers, reactivity in response to psychological stress-induced GC resistance, and a strong increase in IL-6 within 20 min after acute psychological stress, which was followed by a direct impact on peripheral brain function through infiltration from the blood-brain barrier. In contrast, psychologically resilient animals do not show an exacerbated immune response after acute or chronic psychological stress. This may be due to the presence of adaptive mechanisms of GC downregulation of immune system activation in response to psychological stress in mentally resilient individuals (Dudek et al., 2021), for example, mental toughness regulates IL-6 levels by downregulating transcription of Proinflammatory genes and upregulating innate antiviral and antibody-related genes (Gialluisi et al., 2020).

## 5 Possible mechanisms by which physical exercise ameliorates stress-mediated NDDs

Chronic psychological stress that is beyond an individual's tolerance can trigger a range of pathological and physiological responses, increasing that individual's susceptibility to NDDs, exacerbating the condition, and/or accelerating the course of the disease. While, high levels of psychological resilience may be a protective factor for CNS health, protecting mental and physical health and reducing chronic diseases such as NDDs (Kusz and Ahmad, 2020). Mental toughness is not constant, it can be strengthened, helping to enhance the alleviation of NDDs, and anyone can increase their mental toughness to alleviate NDDs, and accelerate and facilitate recovery from NDDs (Babić et al., 2020). Physical activity is the key to keeping high mental toughness and is accessible to all (Lou and Yan, 2020). It can improve mental toughness, and maintain or improve brain function by increasing mental toughness (Chow et al., 2022). It is an effective and low-cost non-pharmacological strategy to prevent or delay chronic stress-mediated NDDs and to maintain physical health and a good quality of life (Chow et al., 2022; Nowacka-Chmielewska et al., 2022). Dunston et al. (2022) and Yu and Ye (2023) demonstrated that people who engage in a large amount of strenuous physical activity have a higher level of mental toughness. Moreover, it was found that the relationship between mental toughness and physical activity may be bi-directional; individuals who engage in regular physical activity seem to have better mental toughness, and individuals with better mental toughness are more likely to be more motivated and confident to engage in physical activity (Yu and Ye, 2023), thus creating a virtuous cycle of improvement in NDDs disease.

Physical activity enhances mental toughness, which may be because regular physical activity leads to positive physiological and psychological benefits, such as improved recognition, mood in patients with NDDs (Kusz and Ahmad, 2020) and stress resilience (Nowacka-Chmielewska et al., 2022), as well as improved physiological control in physical activity in response to psychological stress (Hamer, 2012), Preventing the possible adverse effects of stressful events and preventing many chronic diseases such as NDDs (Deuster and Silverman, 2013). The stress-buffering effects of physical activity play an important role in the amelioration of stress-mediated NDDs by physical activity (Hamer, 2012), which may be related to the psychological and neurophysiological mechanisms mediated by physical exercise.

### 5.1 Psychological mechanisms

Regular physical activity increases resilience and mental toughness and reduces stress vulnerability. Evidence from human and animal studies suggests that a sedentary lifestyle is associated with stress vulnerability, whereas a lifestyle of regular physical activity is associated with stress resilience and mental toughness (Nowacka-Chmielewska et al., 2022).

**Figure 4:**
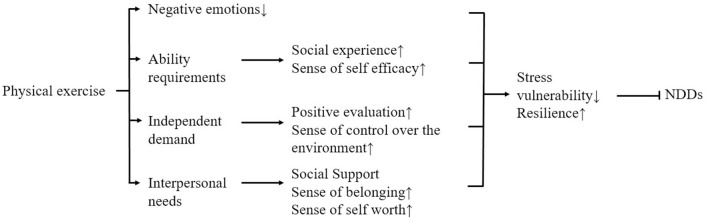
Possible psychological mechanisms of how physical exercise improves NDDs mediated by psychological stress.

In addition, physical activity can enhance psychological resilience and improve mental health by alleviating negative emotions and mediating the need for competence, autonomy, and relationships (Yu and Ye, 2023) to prevent stress-mediated NDDs (Figure 4).

Satisfaction of the need for competence mediated most strongly between physical activity and psychological resilience. High perceptions of competence promote positive expectations of achievement behaviors, which enable people to cope with stress confidently and positively, thereby increasing psychological resilience and mental toughness. Physical activity is positively related to the satisfaction of competence needs, and active participation in physical activity can satisfy college students' perceptions of competence and self-efficacy (Doré et al., 2020) because high levels of competence can be satisfied during physical activity. Deci and Ryan (2000) suggest that this may be because the fulfillment of competence needs occurs in high-quality social experiences, and participation in physical activity provides access to these social experiences, such as cooperation, obtaining feedback, and supporting each other's successes, which helps to satisfy participants' competence needs. This leads to increased mental toughness. According to basic psychological needs theory, competence needs satisfaction promotes perceptions of self-efficacy, which maintains high levels of effortful behavior and demonstrates greater psychological resilience.

The fulfillment of autonomy needs mediates relatively little between physical activity and mental toughness. Autonomy needs are the belief that one's behavior is entirely self-selected and controlled by one's own will. It is positively related to mental toughness. Engaging in physical activity, especially unstructured physical activity, facilitates the satisfaction of autonomy needs, and physical activity is positively related to the satisfaction of autonomy needs. This may be because physical activity creates an environment that supports autonomy and allows individuals to view their behavior as self-approving, thus achieving the satisfaction of their need for autonomy. Individuals with fulfilled autonomy needs are more inclined to positive situational assessments and have a higher sense of control over their environment, which can lead to better coping with psychological stress and higher psychological resilience (Curran et al., 2016).

Relationship needs satisfaction mediated the least between physical activity and mental toughness. Relationship needs, a belief that one is connected to and valued by others, is closely related to mental toughness. When a person develops positive and intimate relationships with others, they build a strong social support system to cope with psychological stress. Perceived social support, respect, and understanding contribute to psychological resilience and enable individuals to respond effectively to stressors. Physical activity is positively associated with the fulfillment of relationship needs. Stathi et al. (2002) suggest that this may be because physical activity promotes social interaction and helps them build social support networks, thus providing patients with a greater sense of belonging, fulfilling their relational needs, and giving them more courage and confidence to deal with psychological stressors and NDDs. In addition, in this environment of social interaction provided by sport, the individual feels that he/she is important, facilitating a sense of self-worth and perceived importance, which may enable individuals to cope with the psychological stress of NDDs or to make sustained efforts to move closer to the goal of alleviating NDDs, thereby improving the psychological resilience of people with NDDs (Xu et al., 2021).

### 5.2 Neurophysiological mechanisms

**Figure 5:**
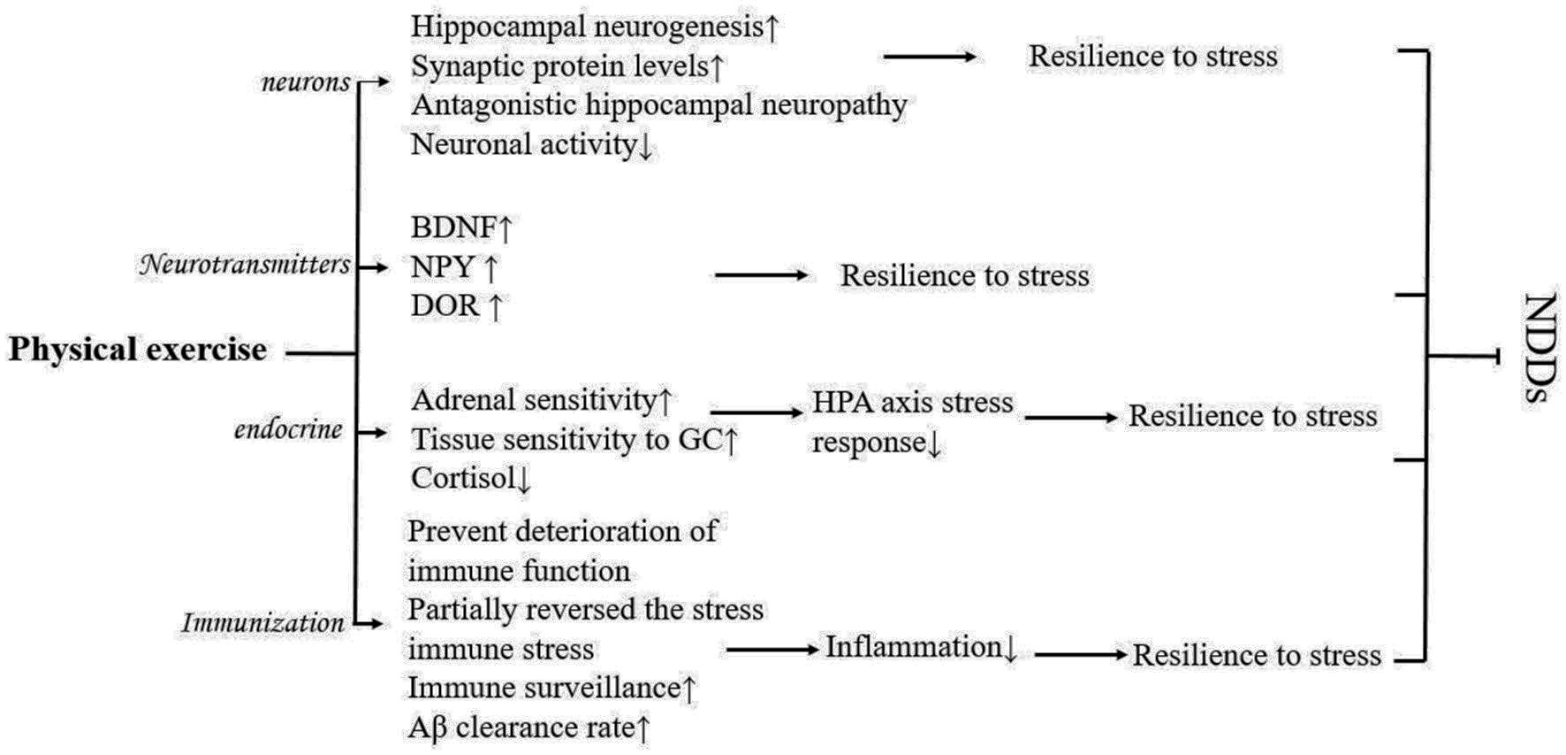
Possible physiological mechanisms of how physical exercise improves NDDs mediated by psychological stress.

Physical exercise may reduce the deleterious effects of psychological stress and improve mood and cognitive functioning. As a prerequisite for exposure to various psychological stressors, regular physical activity fulfills the neuroprotective potential of physical activity by enhancing neurogenesis, and neuroplasticity, increasing the expression of neurotrophic factors and markers of synaptic plasticity, optimizing neuroendocrine and physiological responses such as the HPA axis, and reducing brain inflammation and oxidative stress (Nowacka-Chmielewska et al., 2022) and positively affects the CNS function (Wermelinger Ávila et al., 2018), and adaptive effects on the homeostatic regulation of the system, increasing mental toughness and resistance to psychological stress, and preventing NDDs (Yu and Ye, 2023) (Figure 5).

As one of the strongest physiological modulators of hippocampal structure and function, physical exercise can promote recovery from psychological stress by enhancing hippocampal neurogenesis and increasing synaptic protein levels, thereby reducing the harmful effects of psychological stress (Huang and Zhou, 2021). Stranahan et al. (2007) found that both voluntary and involuntary physical activity increased hippocampal neurogenesis and neuronal cell proliferation in mice. Physical activity may improve cognitive function by minimizing or even reversing the effects of psychological stress on hippocampal neurogenesis. In addition, physical exercise also has the potential to increase neuronal activity by reducing psychological stress (Yu and Ye, 2023) which improves stress-mediated NDDs.

Physical exercise also promotes recovery from psychological stress by increasing the levels of synaptic proteins such as BDNF, thereby reducing the harmful effects of psychological stress (Lehmann and Herkenham, 2011; Zhang et al., 2021a). Animal experiments confirmed this idea, as rats that received 6 weeks of early physical exercise were resistant to psychological stress, showing a trend of decreased anxiety and startle response, as well as an increase in the expression of BDNF, neuropeptide Y (NPY), and delta-opioid receptor (DOR) signaling. It has been suggested that this may be because the increase in BDNF, NPY, and DOR enhances synaptic plasticity, resulting in rats easily recovering from psychological stress states. Recent studies have found that physical exercise can activate the gene encoding peroxisome proliferator-activated receptor γ coactivator 1-alpha (PGC-1α) in human skeletal muscle, inducing an increase in the level of PGC-1α in skeletal muscle, which in turn activates the expression of fibronectin type III domain-containing 5 (FNDC5) (Zhan et al., 2018), activation of the PGC-1α/FNDC5 pathway can induce BDNF to bind Tropomyosin receptor kinase B (TrkB) receptors to improve adaptation to psychological stress (Nowacka-Chmielewska et al., 2022). Running experiments in rats revealed that the deleterious effects of exposure to psychological stress can be prevented by activating the BDNF pathway through early physical exercise (Nasrallah et al., 2019), producing a favorable preventive effect of NDDs and reducing susceptibility to NDDs. However, there is controversy over the type of physical activity that increases BDNF (Chow et al., 2022).

Stress-resistant individuals are characterized by their ability to resist psychological stress responses, low glucocorticoid production during psychological stress, and high physical activity. Physical activity may also alter biological stress responses such as cortisol, markers of inflammation (Hamer, 2012) that produce anti-stress effects. Treadmill exercise has been found to make animals resistant to acute psychological stress and subsequent increased HPA axis effects through experiments in mice (Yuede et al., 2018), to improve resistance (Pedrinolla et al., 2017) and their pathological changes. Physical exercise has beneficial effects on HPA axis function possibly through increased adrenal sensitivity, tissue sensitivity to GC, and reduced cortisol response. However, although physical exercise-induced reductions in cortisol have been documented, researchers have not yet been able to replicate the results of this study (Hamer, 2012).

Acute psychological stress causes an increase in inflammatory markers such as IL-6 and tumor necrosis factor alpha (TNF-a), and physical activity has potential anti-inflammatory effects. Physical exercise can prevent the degradation of immune system function (Lou and Yan, 2020; Qiu et al., 2023) and even partially reverse stress-induced changes in immune function (Rajkumar, 2021) and thus reduce inflammation. Studies have shown that long-term regular physical activity is negatively associated with various Proinflammatory markers (Hamer, 2007). The following are some of the key factors that may contribute to the reduction of inflammation. Long-term moderate-intensity physical activity can benefit the immune system by enhancing immune surveillance and immunocompetence. Regular physical activity improves anti-inflammatory capacity. For example, after 12 weeks of combined moderate-intensity and endurance physical activity, the number of inflammatory CD14 and CD16 monocytes, and Proinflammatory monocyte subtypes were reduced (Timmerman et al., 2008). Fleshner (2005) found that 6 weeks of physical activity was effective in buffering the effects of stress on the immune system, reducing inflammatory cytokines, anxiety-related behaviors, and social avoidance in a rat wheel-running experiment. Both voluntary and mandatory rotational running can prevent stress-induced behavioral changes in rats, and plasticity in brain regions associated with dopamine reward is enhanced, these plasticity changes contribute to resistance to psychological stress, which collectively counteracts CNS inflammation or other negative physiological effects, ultimately leading to protection or slowing of disease progression of NDDs (Yuede et al., 2018).

However, current physical activity trials have not always been consistent in their results regarding the reduction of inflammatory markers after physical activity interventions, with Proinflammatory cytokines, oxidative stress, and cortisol following acute physical activity (Cavalcante et al., 2017; Pedersen, 2011) may increase. However, acute physical activity also beneficially affects immune health, possibly because acute physical activity transiently mobilizes immune cells to peripheral tissues for immune surveillance (Qiu et al., 2023). Moreover, the cytokines released during acute physical exercise originate from physical exercise in skeletal muscle, which is different from IL-6 released during acute psychological stress, and the release of exercise factors from skeletal muscle may take place through signaling pathways, which can have specific endocrine effects on various organs and signaling pathways. At the same time, intramuscular IL-6 expression is regulated by a network of signaling cascades that may involve the CA2+/NFAT and glycogen/p38MAPK pathways. Therefore, acute physical exercise-induced IL-6 release is not a potent Proinflammatory agent (Hamer, 2007).

High cognitive performance in adversity positively influences responses to stress and facilitates emotional stabilization (Kusz and Ahmad, 2020). Whereas acute psychological stress can have an impact on extracellular Aβ levels, a large accumulation of Aβ can lead to neuronal histological degeneration, impaired neuroglial stress, and cognitive decline (Li et al., 2022; Zhang et al., 2023), leading to a vicious circle, which leads to a vicious cycle. Physical activity is associated with physical, cognitive, and emotional resilience (Kusz and Ahmad, 2020). In mice, it was found that Aβ levels in physically active mice were much less affected by acute psychological stress than in sedentary mice. This may be because physical activity can have a positive effect on the onset and progression of NDDs by improving Aβ clearance. Whether physical exercise increases the Aβ clearance pathway and alters gene expression in the hippocampus to increase resistance to psychological stress in mice needs further investigation (Yuede et al., 2018).

## 6 Effectiveness of different types of physical exercise in improving stress-mediated NDDs

Individuals respond differently to stress may be due to individual differences in baseline physical activity, and in animal experiments, mice with high baseline physical activity showed resistance to chronic social stress compared to animals that neglected physical exercise (Zhang et al., 2021b). It is known that Physical activity influences psychological stress, psychological resilience, and NDDs. But differences in the intensity and duration of physical activity, as well as the amount of psychological stress perceived by the type of physical activity (forced or voluntary, solo or group, aerobic or anaerobic, etc.), may play a role to varying degrees in conferring psychological resilience, resisting psychological stress, or in positively influencing physiological mechanisms (Yuede et al., 2018). By reviewing animal as well as human studies, it was found that the psychological stress resistance corresponding to different physical exercises, the ability to prevent NDDs (Table 1) and give recommendations accordingly.

**Table 1:** A Comparative analysis of different PE in improvement of resilience and improvement of NDDs.

**References**	**Type of physical activity**	**Type of study**	**Subject**	**Number of research subjects**	**Study finds**
Nowacka-Chmielewska et al. (2022)	Medium intensity	Poll	University student	244	Mental toughness did not improve with increasing moderate-intensity physical activity.
Wilkaniec et al. (2021)	Medium intensity	Meta- analysis		90,471	Moderate-intensity physical activity can have a beneficial effect on NDDs by facilitating multiple physiological adaptations, thereby reducing anxiety responses.
Babić et al. (2020)	Long-term medium intensity	Literature review			Long-term moderate-intensity physical activity can benefit the immune system by enhancing immune surveillance and immunity.
Nowacka-Chmielewska et al. (2022)	High strength	poll	University student	244	Vigorous physical activity and mental toughness were positively correlated.
Babić et al. (2020)	Rigorous physical exercise	Literature review			In physically inactive individuals, sudden strenuous physical activity can lead to adverse cardiovascular events, and prolonged strenuous physical activity may increase the incidence of infarction.
Nowacka-Chmielewska et al. (2022)	Excess	Poll	University student	244	Physical exercise may only lead to favorable psychosocial factors for college students.
Babić et al. (2020)	Physical activity in a single session	Literature review			A single amount of physical activity may disturb the body's balance.
Nieman and Pence (2020)	Short-term physical exercise	Randomized control	Female (age 28.7 years ± 6.1 years)	40	Short-term physical activity may not be sufficient to induce a stress-buffering effect.
Tuan et al. (2024)	Long-term physical activity	Randomized control	Adult male rats	36	Long-term and regular physical activity has neuroprotective properties against neurodegenerative diseases.
Chow et al. (2022)	Regular/regular physical activity	Meta- analysis		30	Regular or disciplined physical activity positively impacts CNS function, helping to improve an individual's mood and cognitive abilities such as memory and learning, helping to increase the expression of BDNF and markers of synaptic plasticity, and reducing inflammatory factors.
Watanabe and Takeda (2022)	Group exercise	Literature review			Group physical activity may have better results and be more conducive to overall mental toughness than solo physical activity.
Tuan et al. (2024)	Aerobic exercise	Cross- sectional studies	Older people	312	Aerobic physical activity helps to combat the negative consequences of psychological stress and contributes to mental toughness.
Gialluisi et al. (2020)	Voluntary/compulsory forms	Randomized control	Young adult rats	47	Both voluntary and mandatory forms can help prevent stress-induced behavioral changes.

The amount of physical activity is probably the most important of all factors influencing mental toughness (Dunston et al., 2022). Research has shown that vigorous physical activity and a sedentary lifestyle are closely related to physical and psychological health (Yu and Ye, 2023). Besides, vigorous physical activity and mental toughness are positively correlated, with higher levels of mental toughness in those who engage in at least 75 min of vigorous physical activity every week, and no improvement in mental toughness with increasing moderate physical activity (Dunston et al., 2022). However, both preclinical and clinical studies have shown that moderate-intensity physical activity can have a beneficial impact on NDDs by facilitating multiple physiological adaptations, thereby reducing anxiety responses (Anderson and Shivakumar, 2013; Wegner et al., 2014). Prolonged moderate-intensity physical activity can benefit the immune system by enhancing immune surveillance and immunocompetence (Nieman and Pence, 2020). Secondly, physical activity is highly dependent on physical activity intensity in improving Aβ clearance (Yuede et al., 2018). In physically unexercised individuals, sudden strenuous physical activity can lead to adverse cardiovascular events, and prolonged strenuous physical activity may increase the incidence of infarction. Therefore, the intensity and modality of physical activity are crucial to have a positive impact on health (Qiu et al., 2023).

Differences in the duration of physical activity can also affect stress resistance effects. Acute exercise may be insufficient for alleviating stress or enhancing resilience in participants (Tuan et al., 2024). Although acute exercise, such as acute aerobic or resistance exercise, can transiently elevate circulating BDNF (Dinoff et al., 2017), existing experimental findings regarding acute exercise-induced increases in hippocampal BDNF in rats are inconsistent (Takimoto and Hamada, 2014; Sheikhzadeh et al., 2015). Therefore, whether acute exercise elevates hippocampal BDNF in NDD patients remains inconclusive. Furthermore, increases in inflammatory markers like IL-6 in cerebrospinal fluid have been observed following acute vigorous exercise (Steensberg et al., 2006), which could be detrimental for NDD patients. In summary, the benefits of acute exercise for NDD patients appear limited. Short-term physical activity may not be sufficient to induce stress-buffering effects because individual changes in fitness are usually modest, so a short 2-week exercise withdrawal period is insufficient to see changes (Hamer, 2012; Poole et al., 2011). Whereas long-term and regular physical activity has neuroprotective properties against NDDs (Hoffman et al., 2016; Nowacka-Chmielewska et al., 2022). This may be because long-term physical activity can adaptively affect the homeostatic regulation of the system and prevent immune senescence, thereby increasing mental toughness and physical resilience to prevent NDDs (Yuede et al., 2018), and long-term physical activity upregulates BDNF in the hippocampus, enhancing hippocampal synaptic plasticity, neurogenesis, and memory function. For example, after long-term treadmill physical activity, researchers found newly formed synapses in cortical areas (Chen et al., 2017, 2019). PGC-1α levels are higher in muscles with long-term physical activity, so people with longer levels of habitual physical activity may have more significant effects (Hamer, 2012). Regular physical activity positively affects CNS function, helping to improve an individual's mood and cognitive abilities such as memory and learning, helping to increase the expression of BDNF and markers of synaptic plasticity, and reducing inflammatory factors (Nowacka-Chmielewska et al., 2022), especially if one already has NDDs (Qiu et al., 2023). This stress-buffering effect of regular physical activity is long-term, with a person who exercises three times a week achieving 12 hours of stress-buffering over a week (Lou and Liu, 2018; Hamer, 2012), whereas a single session of physical activity may disrupt homeostasis in the body (Nieman and Pence, 2020). Therefore, long-term chronic physical activities and regular physical activities may help the body adapt to psychological and physical stress more than short-term and single-session physical activity (Yuede et al., 2018). Furthermore, research has found that 650 MET-min/week is the optimal exercise dosage for maximal benefits (Yuan et al., 2024).

Group physical activity may have better results than solo physical activity. Participation in group-based sporting events or organized charity events is more conducive to improving overall mental toughness as it increases the opportunity to improve physiological toughness while engaging in social interactions (Kusz and Ahmad, 2020). Yoga and Tai Chi are classified as mind-body exercises. Evidence suggests these modalities offer superior benefits by integrating cognitive engagement with exercise, leading to: (1) enhanced balance, coordination, gait, and agility; (2) structural improvements in neuronal networks, synaptic plasticity, and cerebrovascular systems; (3) strengthened connectivity across brain regions, thereby augmenting executive function, memory consolidation, and emotional regulation; and (4) suppression of neuroinflammation. This multimodal neuroprotection makes them particularly targeted for AD with memory decline and cognitive impairment. Dance demonstrates significant advantages in stress reduction. As a dual-task activity requiring simultaneous motor execution and step memorization, it concurrently improves motor function and cognitive processing. Rapid step transitions during improvisational dance further enhance cognitive acuity beyond Yoga/Tai Chi. Crucially, dance incorporates musical accompaniment, while partnered styles necessitate verbal communication, creating unique opportunities for improving verbal fluency and language proficiency. Mounting evidence confirms dance effectively attenuates psychological stress and elevates cognitive sharpness (Rao et al., 2023).

Aerobic exercise helps to combat the negative consequences of psychological stress and contributes to mental toughness (Wermelinger Ávila et al., 2018). In particular, aerobic exercise also improves memory and cognitive functioning (Windle et al., 2010). For example, swimming reduced vulnerability to chronic mild psychological stress in mice and contributed to neuroprotective effects mediated by the AKT/GSK-3β/CRMP2 pathway and microtubulenducing depress dynamics. Five weeks of swimming exercise also prevented chronic mild psychological stress from ion-like behavior in mice (Sun et al., 2021). Therefore, swimming exercise is a good physical activity to improve resistance to psychological stress. It has also been found in rat experiments that both voluntary and mandatory forms help prevent psychological stress-induced behavioral changes (Herrera et al., 2016; Yuede et al., 2018).

It has been suggested that interrupting physical activity can cause mood disturbances, which can become a mild inflammatory stimulus, negatively impacting mental toughness and NDDs. However, recent studies have found no inflammatory markers such as IL-6, C-reactive protein (CRP), and TNF-α even after successfully inducing increased negative mood after weeks of interrupted physical activity. However, periods of physical activity withdrawal may lead to increased cortisol responsiveness to psychological stressors (Hamer, 2012).

Individual differences could influence the effectiveness of exercise in building resilience and slowing NDD progression. Physical exercise demonstrates superior efficacy only in mild-to-moderate neurodegenerative disorders. Consequently, its therapeutic benefits may be limited in patients with rapid disease progression or advanced-stage conditions. High-intensity exercise yields optimal outcomes. However, exercise is contraindicated in individuals with absolute contraindications such as pressure ulcers or acute infections, precluding its prescription as a therapeutic modality. Evidence confirms that psychosocial factors—particularly social support and exercise volition—could modulate resilience (Chen et al., 2022; Neuner et al., 2022). Individuals with strong social support are more likely to have high resilience. Those with family support or healthy social connections tend to be more confident in enhancing their resilience. Individuals with high exercise motivation can achieve better fitness outcomes, meaning active exercise improves resilience more effectively than passive exercise, helping them combat NDDs.

Consequently, we propose a combined prescription: (1) ≥75 min of high-intensity exercise weekly, (2) distributed across ≥3 sessions, (3) achieving total energy expenditure of 650 MET-min/week. To enhance exercise adherence, group-based high-intensity interval training (HIIT) is recommended.

## 7 Conclusion

It has been theorized and practiced that oxidative stress, mitochondrial dysfunction, excessive glutamate build-up, and long-term chronic inflammation lead to susceptibility to NDDs, increased severity of NDDs, and delay in the course of NDDs. In recent years, it has been realized that psychological factors such as psychological stress and mental toughness can also have a negative or positive impact on the development of NDDs. Psychological stress can negatively affect NDDs through endocrine and immuno-inflammatory mechanisms, while psychological resilience can have a positive effect on NDDs through psychological and neurobiological mechanisms. At the neurophysiological level, physical activity can have neuroprotective potential by reducing CNS inflammation and oxidative stress through processes such as enhancing neurogenesis, and neuroplasticity, increasing neurotrophic factors, and attenuating the HPA axis stress response. On a psychological level, physical activity can enhance people's psychological resilience by reducing negative emotions and through the mediation of competence needs, autonomy needs, and relationship needs. For patients with NDDs, physical activity can prevent the onset of NDDs, alleviate symptoms, and/or shorten the duration of the disease by improving patients' psychological resilience through the aforementioned mechanisms of resisting the negative stress effects of psychological stress. Physical exercise has shown potential theoretical significance and applied value for NDDs, but the current direct evidence is not sufficient to fully describe its mechanisms and effects. Further research can be conducted in the future using a more rigorous experimental design and developing scientifically based exercise prescriptions based on the stress response system practices of psychological stress and mental toughness.

### 7.1 Vantage and limitations

Vantage: Theoretically, this study offers some innovation by creatively integrating exercise, psychological stress/resilience, and NDDs pathological mechanisms, providing a detailed mechanistic explanation; It establishes an important theoretical framework and generates hypotheses for understanding the “psychogenic” pathways through which exercise improves NDDs; It compares different exercise durations, frequencies, intensities, and modalities, with a focus on practical applications. Limitations: Only qualitative analysis was conducted, without quantitative methods such as meta-analysis or network meta-analysis; No longitudinal intervention studies (e.g., randomized controlled trials) were included, and high-level direct evidence in NDDs populations is still lacking; Challenges in clinical generalizability and translation require further investigation; Final conclusions and clinical applications need more rigorous intervention studies targeting NDDs patients, ideally using controlled experimental designs to directly test the full mediation model (Al-Ali et al., 2024).
